# Impact of Extrinsic and Intrinsic Hypoxia on Catecholamine Biosynthesis in Absence or Presence of Hif2α in Pheochromocytoma Cells

**DOI:** 10.3390/cancers11050594

**Published:** 2019-04-28

**Authors:** Nicole Bechmann, Isabel Poser, Verena Seifert, Christian Greunke, Martin Ullrich, Nan Qin, Axel Walch, Mirko Peitzsch, Mercedes Robledo, Karel Pacak, Jens Pietzsch, Susan Richter, Graeme Eisenhofer

**Affiliations:** 1 Institute of Clinical Chemistry and Laboratory Medicine, University Hospital Carl Gustav Carus, Technische Universität Dresden, Fetscherstrasse 74, 01307 Dresden, Germany; Isabel.poser@uniklinikum-dresden.de (I.P.); Mirko.peitzsch@uniklinikum-dresden.de (M.P.); susan.richter@uniklinikum-dresden.de (S.R.); Graeme.eisenhofer@uniklinikum-dresden.de (G.E.); 2 Department of Radiopharmaceutical and Chemical Biology, Helmholtz-Zentrum Dresden-Rossendorf, Institute of Radiopharmaceutical Cancer Research, Bautzner Landstrasse 400, 01328 Dresden, Germany; v.seifert@hzdr.de (V.S.); m.ullrich@hzdr.de (M.U.); j.pietzsch@hzdr.de (J.P.); 3 Research Unit Analytical Pathology, Helmholtz Zentrum München, German Research Center for Environmental Health (GmbH), Neuherberg, Germany; Ingolstädter Landstraße 1, 85764 Neuherberg, Germany; christian.greunke@gmx.de (C.G.); axel.walch@helmholtz-muenchen.de (A.W.); 4 Division of Pediatric Neuro-Oncogenomics, German Cancer Research Center (DKFZ), 69120 Heidelberg, Germany; nan.qin@med.uni-duesseldorf.de; 5 German Consortium for Translational Cancer Research (DKTK), partner site Essen/Düsseldorf, 45147 Düsseldorf, Germany; 6 Department of Pediatric Oncology, Hematology, and Clinical Immunology, Medical Faculty, University Hospital Düsseldorf, 40225 Düsseldorf, Germany; 7 Department of Neuropathology, Medical Faculty, Heinrich-Heine University Düsseldorf, 40225 Düsseldorf, Germany; 8 Hereditary Endocrine Cancer Group, CNIO, Madrid, Spain and Centro de Investigación Biomédica en Red de Enfermedades Raras (CIBERER), 28029 Madrid, Spain; mrobledo@cnio.es; 9 Section on Medical Neuroendocrinology, Eunice Kennedy Shriver National Institute of Child Health and Human Development, National Institutes of Health, Bethesda, MD 20892, USA; karel@mail.nih.gov; 10 Department of Chemistry and Food Chemistry, School of Science, Technische Universität Dresden, Mommsenstrasse 9, 01062 Dresden, Germany; 11 Department of Medicine III, University Hospital Carl Gustav Carus, Technische Universität Dresden, Fetscherstrasse 74, 01307 Dresden, Germany

**Keywords:** hypoxia, pseudohypoxia, spheroids, HIF, EPAS1, catecholamine, pheochromocytoma and paraganglioma, phosphorylation tyrosine hydroxylase

## Abstract

Pheochromocytomas and paragangliomas (PPGLs) with activated pseudohypoxic pathways are associated with an immature catecholamine phenotype and carry a higher risk for metastasis. For improved understanding of the underlying mechanisms we investigated the impact of hypoxia and pseudohypoxia on catecholamine biosynthesis in pheochromocytoma cells naturally lacking *Hif2α* (MPC and MTT) or expressing both *Hif1α* and *Hif2α* (PC12). Cultivation under extrinsic hypoxia or in spheroid culture (intrinsic hypoxia) increased cellular dopamine and norepinephrine contents in all cell lines. To distinguish further between *Hif1α-* and *Hif2α*-driven effects we expressed *Hif2α* in MTT and MPC-mCherry cells (naturally lacking *Hif2α*). Presence of *Hif2α* resulted in similarly increased cellular dopamine and norepinephrine under hypoxia as in the control cells. Furthermore, hypoxia resulted in enhanced phosphorylation of tyrosine hydroxylase (TH). A specific knockdown of *Hif1α* in PC12 diminished these effects. Pseudohypoxic conditions, simulated by expression of *Hif2α* under normoxia resulted in increased TH phosphorylation, further stimulated by extrinsic hypoxia. Correlations with PPGL tissue data led us to conclude that catecholamine biosynthesis under hypoxia is mainly mediated through increased phosphorylation of TH, regulated as a short-term response (24–48 h) by HIF1α. Continuous activation of hypoxia-related genes under pseudohypoxia leads to a HIF2α-mediated phosphorylation of TH (permanent status).

## 1. Introduction

Pheochromocytomas and extra-adrenal paragangliomas (PPGLs) are rare catecholamine-producing neuroendocrine tumors with variable aggressiveness. PPGLs with activated pseudohypoxic pathways (cluster 1), including those with mutations in genes encoding hypoxia-inducible factor (*HIF*) *2α* (also known as *EPAS1*), von Hippel-Lindau tumor suppressor (*VHL*), prolyl hydroxylase domain (*PHD*), fumarate hydratase, and succinate dehydrogenase subunits (*SDHx*) are characterized by an immature catecholamine phenotype and higher risk of metastasis particularly prevalent in SDHx-mutated tumors [1,2,3]. In contrast, PPGLs with genetic alterations associated with activated kinase signaling pathways (cluster 2) are mostly benign and show a mature catecholamine phenotype with strong expression of phenylethanolamine *N*-methyltransferase (PNMT), the enzyme that converts norepinephrine (NEpi) to epinephrine (Epi) [3,4]. Increased stabilization of HIFs and resulting activation of hypoxia-related pathways seem to play a central role in the development and progression of PPGLs [5]. Under normoxic conditions, the oxygen-sensitive HIFα subunit of the HIFα/HIFβ complex is degraded by PHD- and VHL-mediated mechanisms. Insufficient oxygen (≤1% oxygen), also known as hypoxia, leads to stabilization of the HIFα subunit resulting in the regulation of numerous HIF-target genes, either by interaction with HIF1β followed by the transactivation of the hypoxia responsive element (HRE) or by interactions with NOTCH, WNT, and MYC pathways [6].

There are two main HIFα isoforms, HIF1α and HIF2α, with partly overlapping functions, regulating numerous processes including angiogenesis, cell survival, stem cell self-renewal and pro-metastatic features of tumor cells. Gene expression profiling and immunohistochemical studies have established enhanced expression of HIF2α, but no differences in expression of HIF1α, in cluster 1 compared to cluster 2 PPGLs [4,7,8,9]. In chromaffin cells, HIF2α seems to be responsible for maintaining the balance between differentiation and stemness of the sympathoadrenal lineage, with both HIFα subunits regulating biosynthesis, storage and secretion of catecholamines [10]. Expression of tyrosine hydroxylase (TH), the rate-limiting enzyme in catecholamine biosynthesis and responsible for conversion of tyrosine to _L_-dihydroxyphenylalanine (L-DOPA), is inducible by hypoxia. Both HIFα subunits are able to bind at the HRE of the *TH* promoter, thereby increasing TH expression [11]. Besides altered expression, TH enzyme activity further depends on posttranslational phosphorylation at serine 8, 19, 31, and 40 [12,13]. A specific knockdown of *Hif2α* by RNA interference had no effect on *Th* mRNA expression in a rat adrenomedullary chromaffin cell line; in contrast, an influence on DOPA decarboxylase (*Ddc*), the enzyme responsible for conversion of L-DOPA to dopamine (DA), was established by changes in mRNA expression [14]. Park and coworkers investigated impacts of HIF1α protein stabilization on catecholamine-induced expression of vascular endothelial growth factor (VEGF) and showed that treatment with NEpi stimulated HIF1α protein stabilization associated with increased angiogenesis [15]. Furthermore, PNMT expression seemed to be predominantly regulated by HIF1α [16,17]. These findings suggest that HIF1α is the major player for the stress-induced fight-or-flight response (short-term response) by regulating the biosynthesis of Epi. In contrast, HIF2α seems to be more important for the regulation of developmental processes (long-term response), such as those associated with immature chromaffin cell features including absence of PNMT expression with missing production of Epi in cluster 1 PPGLs [18,19] and promotes an aggressive phenotype [20].

**Figure 1:**
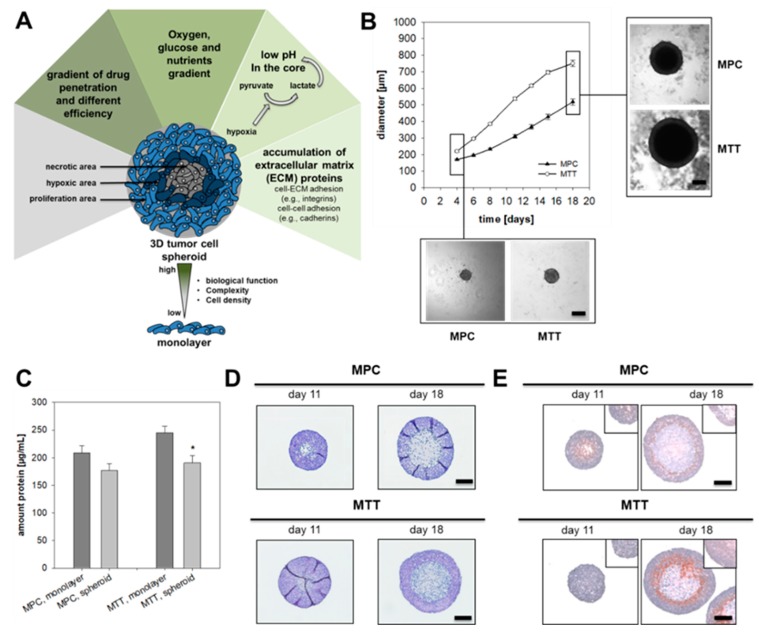
Characterization of pheochromocytoma cell spheroids. (**A**) Tumor cell spheroids are characterized by a necrotic core surrounded by hypoxic cell layers and an external zone of proliferating cells. This structure encourages the formation of an oxygen, pH and nutrient gradient and leads to accumulation of extracellular matrix proteins. Complexity and comparability with the structure of a metastasis offers an important tool for drug screening. (**B**) Growth pattern of MPC and MTT cell spheroids generated by using methylcellulose method. Four independent experiments (*n* = 15–20). Mean ± SEM. (**C**) Impact of spheroid cultivation on the amount of protein produced by 500 cells over a time-period of eight days in comparison to monolayer conditions. Four independent experiments (*n* = 16). Mean ± SEM. ANOVA and Bonferroni post hoc test comparison vs. monolayer, * *p* < 0.05. (**D**) Representative section of pheochromocytoma cell spheroids stained with Hematoxylin and Eosin (nuclei: blue, cytosol: violet). (**E**) Covalent binding of pimonidazole confirmed the development of a hypoxic region (red) surrounding the necrotic core of the spheroids (nuclei: blue). Scale bar: 200 µm.

Three-dimensional tumor cell spheroids provide an excellent in vitro model to study the influence of hypoxia (intrinsic hypoxia) under conditions close to the in vivo situation within tumors. This is because in contrast to monolayer culture, tumor cell spheroids mimic the tumor microenvironment and the structure of the spheroid encourages the formation of an oxygen and nutrient gradient (Figure 1A).

Several studies have shown the excellent suitability of this model for drug screenings [21,22] and investigations of the microenvironment [23] also on pheochromocytoma cell lines. The present study investigates the hypothesis, that pheochromocytoma cell spheroids provide a suitable model to examine chromaffin cell features such as catecholamine biosynthesis in vitro. Therefore, mouse pheochromocytoma cells (MPC) generated from a neurofibromin 1 knockout mouse model [24] and its more aggressive derivate, the MTT cell line [25], were used as models and cultivated under intrinsic or extrinsic (monolayer culture with 1% oxygen) hypoxia. Cellular catecholamine contents were analyzed as a reflection of catecholamine biosynthesis, storage and turnover. We further addressed the question, about whether HIF1α or HIF2α is the key regulator of TH biosynthesis under hypoxic and pseudohypoxic conditions. Specific knockin or knockdown models were utilized to answer this question and in vitro data were compared to gene expression in PPGL tumor tissue.

## 2. Results

### 2.1. Spheroid Growth Pattern and Characteristics

In accordance with growth in monolayer culture, MTT cells showed an enhanced growth pattern in spheroid culture compared to MPC cells (Figure 1B). MPC cell spheroids reached a diameter of approximately 550 µm after 18 days in culture, whereas the MTT cell spheroids already achieved a diameter of 600 µm after 14 days. For both cell lines an optimized cell number (Figure S3) of 500 cells per well were used for spheroid generation to reach an exponential growth pattern over 18 days. Cultivation under spheroid conditions diminished protein contents, an expected finding due to reduced nutrient supply within spheroids (Figure 1C). In comparison to other methods (Figure S3–S5) the use of methyl cellulose leads to uniform spheroids without verifiable outgrowth. Pheochromocytoma spheroids were characterized by a necrotic core surrounded by a narrow hypoxic zone and an external zone of proliferating cells as confirmed by the covalent binding of pimonidazole (Figure 1D,E). MALDI mass spectrometry imaging (MALDI-MSI) was used to analyze the distribution of phosphatidylinositol (PIP) within the spheroids. Higher contents in the proliferating cell layers indicated that the membrane of the cells remained intact, while PIP contents in the necrotic core were reduced (Figure S7). Higher levels of hexose monophosphate in the outer cell layers of the spheroid indicated an enhanced metabolic activity in the hexose monophosphate shunt (Figure S7).

### 2.2. Impact of Extrinsic and Intrinsic Hypoxia on Catecholamine Biosynthesis

**Figure 2:**
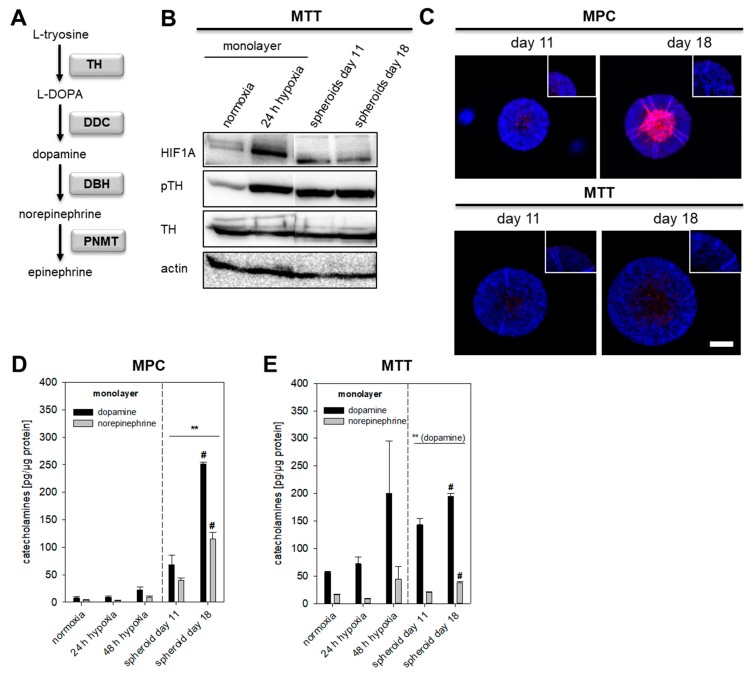
Impact of extrinsic and intrinsic hypoxia on catecholamine biosynthesis. (**A**) Section of biosynthetic pathways of catecholamines. Squares highlighted the underlying enzymes (TH: tyrosine hydroxylase; DDC: DOPA decarboxylase; DBH: dopamine β-hydroxylase; PNMT: phenylethanolamine *N*-methyltransferase). (**B**) Effect of extrinsic and intrinsic hypoxia on the protein expression and phosphorylation of TH at Ser40 in MTT cells. (**C**) Immunohistochemical staining showed an increased expression of TH (red) in the necrotic and hypoxic core of the spheroid (DAPI, blue). Scale bar: 200 µm. Catecholamine content of (**D**) MPC and (**E**) MTT cell spheroids in comparison to monolayer cultivation under normoxic or hypoxic conditions. Three independent experiments (*n* = 3–6). Mean ± SEM. ANOVA and Bonferroni post hoc test comparison vs. normoxia, ** *p* < 0.01, or vs. spheroid day 11, # *p* < 0.05 or, ## *p* < 0.01.

Hypoxia is an important contributor to intra- and inter-tumor cell diversity and is associated with reduced differentiation, as shown in neuroblastoma and breast cancer cells [26,27]. Furthermore, alterations in hypoxia-associated genes in pseudohypoxic cluster 1 PPGLs are associated with an immature catecholamine phenotype [3,18]. The establishment of pheochromocytoma cell spheroids allowed us for the first time to distinguish between short-term effects (extrinsic) and long-term effects (intrinsic) of hypoxia on chromaffin cell characteristics. Long-term exposure to extrinsic hypoxia is not suitable for the currently available pheochromocytoma cell lines, because of the complete loss of cell growth characteristics [28]. We investigated the impact of extrinsic (O_2_ ≤ 1%) and intrinsic hypoxia on catecholamine biosynthesis in several pheochromocytoma cell lines (Figure S6). In MTT cells cultivated under extrinsic hypoxia or spheroid conditions TH protein levels (Figure 2A) were not affected but instead showed increased phosphorylation at Ser40, an indicator of enhanced catalytic activity (Figure 2B). Immunohistochemical staining showed increased expression of TH in the necrotic core and the surrounding hypoxic area of the spheroid (Figure 2C). MPC cells contained lower amounts of basal catecholamines compared to MTT cells. Extrinsic as well as intrinsic hypoxia led to a significant increase of cellular DA in both cell lines (Figure 2D,E). Cultivation under spheroid conditions further increased cellular NEpi. The hypoxic regions and necrotic core of the spheroid enlarged with increasing cultivation time; this was also reflected by enhanced DA and NEpi contents comparing 11- and 18-days old spheroids. Especially in MPC cell spheroids, DA and NEpi were much higher compared to the monolayer culture under extrinsic hypoxia. This indicates an additional impact of necrosis on catecholamine biosynthesis in spheroids.

### 2.3. HIF1α- and HIF2α-Mediated Effects on Catecholamine Biosynthesis

**Figure 3:**
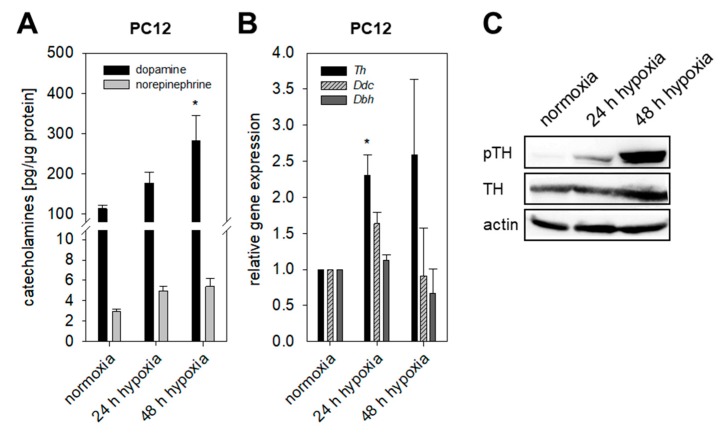
Impact of extrinsic hypoxia in presence of *Hif1α* and *Hif2α*. (**A**) Cellular catecholamines in PC12 cells in dependence of hypoxia. Presence of *Hif1α* and *Hif2α* led to an increase of dopamine within the cells. Three independent experiments (*n* = 6–9). Mean ± SEM. ANOVA and Bonferroni post hoc test comparison vs. normoxia, * *p* < 0.05. (**B**) Hypoxia resulted in a significant up-regulation of *Th* expression in PC12 cells under extrinsic hypoxia, whereas expression of *Ddc* and *Dbh* remained unaffected. Four independent experiments (*n* = 4). Mean ± SEM. ANOVA and Bonferroni post hoc test comparison vs. normoxia, * *p* < 0.05. (**C**) Hypoxia further enhanced phosphorylation of TH at Ser40 investigated by Western blot analysis. Shown is a representative section out of three independent experiments.

MPC and MTT cells naturally lack *Hif2α*. To clarify whether *Hif1α* or *Hif2α* drives the induction of catecholamine biosynthesis under extrinsic hypoxia, we analyzed cellular catecholamines of the rat PC12 cell line expressing both *Hif1α* and *Hif2α*. PC12 cells were unable to form spheroids using different methods for spheroid generation (described in Supplementary Materials). Quantitative real-time polymerase chain reaction (qRT-PCR) showed enhanced expression of *Th* under extrinsic hypoxic conditions, whereas the expression of *Ddc* and *Dbh* remained unchanged (Figure 3B). Cultivation under extrinsic hypoxia led to an increased phosphorylation of TH at Ser40 (Figure 3C) and accumulation of DA in PC12 cells (Figure 3A). Presence of *Hif2α* in PC12 cells seemed not to have any effect on hypoxia-induced catecholamine biosynthesis.

**Figure 4:**
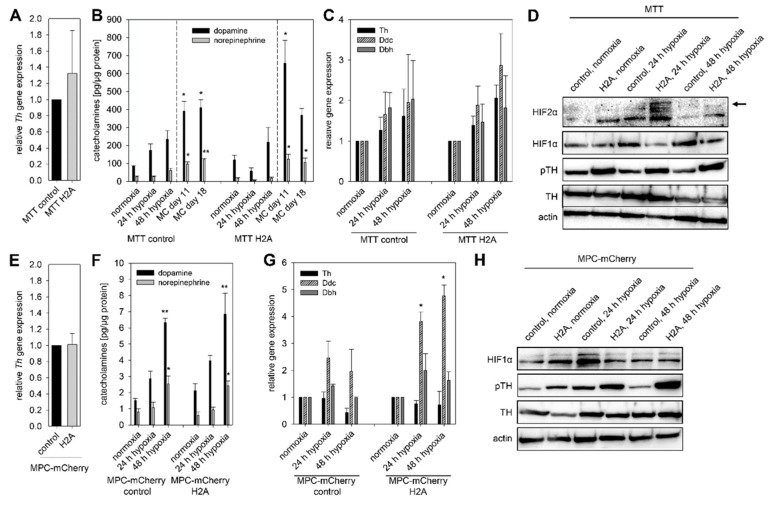
Expression of *Hif2α* in mouse pheochromocytoma cells (pseudohypoxic conditions) enhanced the basal phosphorylation (pseudohypoxic conditions) of TH, which was further increased by the exposure to hypoxia. (**A**) Expression of *Hif2α* in MTT cells had no effect on the relative *Th* expression determined by qRT-PCR. Three independent experiments (*n* = 3). Mean ± SEM. (**B**) In both cell lines with different *Hif2α* expression, exposure to extrinsic or intrinsic hypoxia led to elevated DA and NEpi, especially in spheroids generated with the methyl cellulose (MC) method. Three independent experiments (*n* = 9). Mean ± SEM. ANOVA and Bonferroni post hoc test comparison vs. normoxia, * *p* < 0.05, or ** *p* < 0.01. (**C**) In MTT cells, expression of *Th*, *Ddc* and *Dbh* increased by the exposure to hypoxia independent of their *Hif2α* expression. Three independent experiments (*n* = 3). Mean ± SEM. (**D**) Pseudohypoxia, simulated by the expression of *Hif2α* as well as extrinsic hypoxia led to an enhance phosphorylation of TH at Ser40 in MTT cells. Representative sections of three independent Western blot analysis were shown. To confirm these results MPC-mCherry cells expressing *Hif2α* and their counterpart cell line were used. (**E**) Expression of *Hif2α* in MPC-mCherry cells had no impact on the relative *Th* expression. Six independent experiments (*n* = 6). Mean ± SEM. (**F**) Similar to MTT cells the exposure to hypoxia resulted in enhanced DA and NEpi in both MPC-mCherry cell lines. Three independent experiments (*n* = 9). Mean ± SEM. ANOVA and Bonferroni post hoc test comparison vs. normoxia, * *p* < 0.05, or ** *p* < 0.01. (**G**) Expression of *Hif2α* was associated with increased expression of *Ddc* under extrinsic hypoxia. Four independent experiments (*n* = 4). Mean ± SEM. ANOVA and Bonferroni post hoc test comparison vs. normoxia, * *p* < 0.05. (**H**) Western blot analysis confirmed the enhanced phosphorylation of TH at Ser40 under pseudohypoxic conditions in MPC-mCherry cells. Extrinsic hypoxia further increased this effect. Three independent experiments.

To mimic pseudohypoxic conditions, we expressed codon optimized *Hif2α* in MTT cells (MTT H2A) naturally lacking *Hif2α*. The counterpart cell line, transfected with an empty vector (MTT control), was used as a control (Table S1). The relative *Th* expression (Figure 4A) was not affected by the expression of *Hif2α* (pseudohypoxia). No differences in cellular DA and NEpi content between MTT H2A cells and MTT control cells were observed. Cultivation under extrinsic and intrinsic hypoxia increased cellular DA contents in both cell lines (Figure 4B). Spheroid culture conditions furthermore increased NEpi significantly. The relative expression of *Ddc* and *Dbh* was not affected by the incubation under extrinsic hypoxic conditions (Figure 4C). *Hif2α* expression led to increased phosphorylation of TH while the total amount of TH protein remained unaffected (Figure 4D). In the presence of *Hif2α* (pseudohypoxia), the exposure to extrinsic hypoxia only had a negligible effect on the TH phosphorylation. To confirm the previous results, we used MPC-mCherry cells (no *Hif2α* expression) with expression of *Hif2α* (MPC-mCherry H2A) and their counterpart cell line (MPC-mCherry control) [22]. Similar to the MTT H2A cells, expression of *Hif2α* in MPC-mCherry cells had no effect on *Th* gene expression and basal DA and NEpi contents under normoxic conditions (Figure 4E,F). Independent of *Hif2α* expression, cultivation under hypoxia resulted in increased DA and NEpi contents in both cell lines, indicating a predominantly *Hif1α*-mediated effect under extrinsic hypoxia (Figure 4F). Under extrinsic hypoxia, no effect on *Th* and *Dbh* expression was observed, but the expression of *Ddc* was significantly increased in MPC-mCherry H2A cells (Figure 4G). The expression of *Hif2α* (pseudohypoxia) was accompanied by an enhanced phosphorylation of TH that further increased in both cell lines under extrinsic hypoxia (Figure 4H). The generation of a pseudohypoxic environment by expression of *Hif2α* in MPC and MTT cells permitted comparison of catecholamine biosynthesis, gene expression and TH activation under both extrinsic and intrinsic hypoxia in presence or absence of a pseudohypoxic cellular environment.

**Figure 5:**
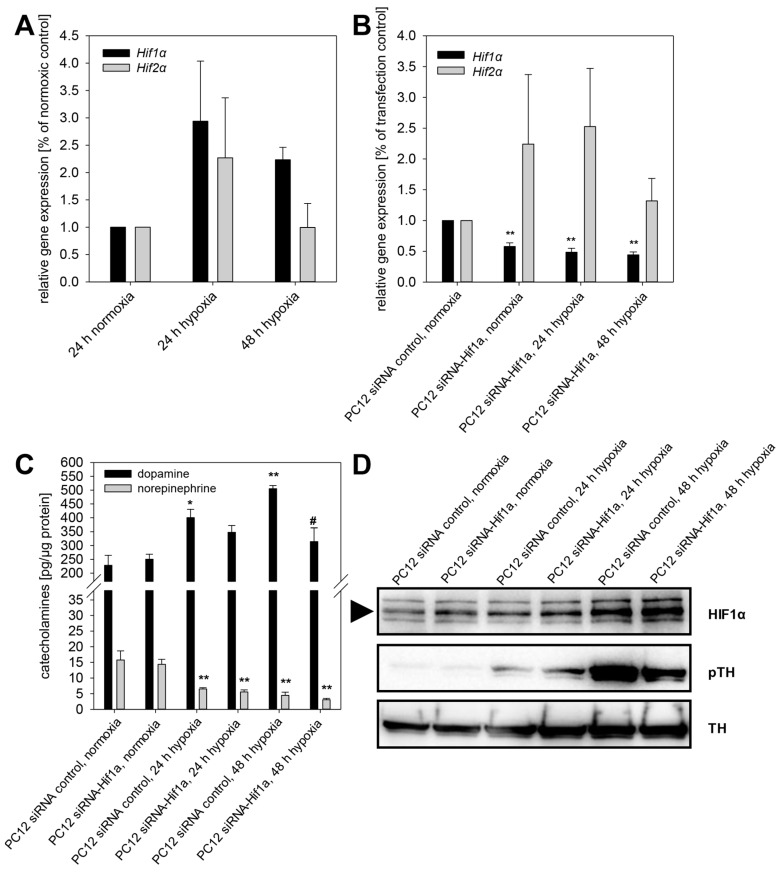
Specific knockdown of *Hif1α* diminished phosphorylation of tyrosine hydroxylase and thereby reduced cellular dopamine content in PC12 cells. (**A**) Extrinsic hypoxia led to a time-dependent upregulation of *Hif1α* and *Hif2α* in PC12. (**B**) RNA interference using siRNA against *Hif1α* repressed the expression of *Hif1α* also under extrinsic hypoxia while *Hif2α* remains unaffected. (**C**) Extrinsic hypoxia increased cellular dopamine content in PC12 control cells (transfection without siRNA). This effect was diminished by knockdown of *Hif1α*. (**D**) Furthermore, phosphorylation of TH was decreased by knockdown of *Hif1α*. Three independent experiments (*n* = 3–6). Mean ± SEM. ANOVA and Bonferroni post hoc test comparison vs. normoxia, * *p* < 0.05 or, ** *p* < 0.01, or vs. PC12 siRNA control 24 or 48 h hypoxia, # *p* < 0.05.

Exposure to extrinsic hypoxia led to a time-dependent upregulation of *Hif1α* and *Hif2α* in PC12 (Figure 5A). In the next step, we reduced the expression of *Hif1α* in PC12 cells using RNA interference. A stable knockdown efficiency of 42.1 ± 5.7% was achieved over at least 72 h, also under extrinsic hypoxia (Figure 5B,C). The knockdown of *Hif1α* had no effect on expression of *Hif2α* (Figure 5B). Specific knockdown of *Hif1α* reduced hypoxia-induced DA content of PC12 cells significantly (Figure 5C). Moreover, specific knockdown of *Hif1α* led to reduced phosphorylation of TH at Ser40, while the total TH protein amount remained unaffected (Figure 5D).

### 2.4. TH Expression under Pseudohypoxic Conditions In Vitro and In Vivo

**Figure 6:**
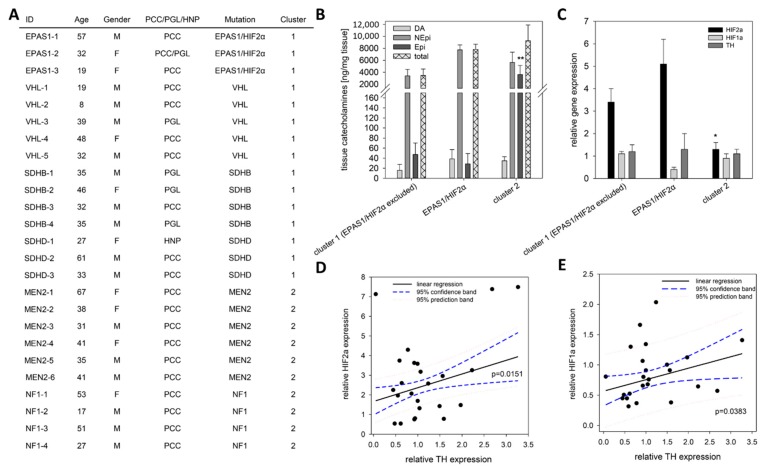
Expression pattern of *EPAS1/HIF2α*, *HIF1α* and *TH* in human tumor tissue. (**A**) Clinical characteristics of the included patients with confirmed mutation in well-described cluster 1 or cluster 2 related genes. (**B**) Tumor tissue from patients carrying a somatic gain-of-function mutation in *EPAS1/HIF2α* (*n* = 3) showed twice as much NEpi than other cluster 1 tumors that is also reflected by the total amount of catecholamines (sum of DA, NEpi and Epi). An elevated content of Epi was observed for mature cluster 2 tumors in comparison to the immature cluster 1 tumors. Mean ± SEM. ANOVA and Bonferroni post hoc test comparison vs. cluster 1 and EPAS1/HIF2α, ** *p* < 0.01. (**C**) qRT-PCR analysis showed an elevated *EPAS1/HIF2α* expression in cluster 1 PPGLs; whereas the *TH* expression remains unaffected by the underlying mutations in the different cluster. Mean ± SEM. ANOVA and Bonferroni post hoc test comparison vs. cluster 1 and EPAS1/HIF2α, * *p* < 0.05. (**D**) A significant linear correlation between the expression of *EPAS1/HIF2α* and *TH* could be detected (f = 1.667 + 0.681x, r = 0.490, R^2^ = 0.2399). (**E**) A similar correlation was also observed for the expression of *HIF1α* and *TH* (f = 0.566 + 0.190x, r = 0.4252, R^2^ = 0.1808).

In contrast to extrinsic and intrinsic hypoxia, pseudohypoxic conditions are characterized by the presence of oxygen with simultaneous activation of hypoxia-related pathways. We simulated pseudohypoxic conditions in vitro by the expression of *Hif2α* in MPC-mCherry and MTT cells under normoxic conditions (Figure 4). Expression of *Hif2α* had no effect on the expression of *Th* and cellular DA and NEpi contents. To correlate these findings with the in vivo situation, the *TH*, *HIF1α* and *HIF2α* expression in tumors from PPGL patients with known genetic mutation (Figure 6A) were analyzed using qRT-PCR (Figure 6C–E). Similar to other pseudohypoxic cluster 1 (5 *VHL*, 4 *SDHB*, 3 *SDHD*) PPGLs, tumors with a somatic gain-of-function mutation in *EPAS1/HIF2α* (*n* = 3) showed an increased expression of *HIF2α* compared to cluster 2 tumors (4 *NF1*, 6 *MEN2*), confirming previous results [4] in the present cohort (Figure 6A,C). All three groups (*EPAS1/HIF2α* vs. cluster 1 vs. cluster 2) showed a similar *TH* expression independent of *HIF2α* expression (Figure 6C), which is in accordance with our in vitro findings using mouse pheochromocytoma cells expressing *Hif2α* (Figure 4A,E). The three patients with *EPAS1/HIF2α* mutation consistently showed a doubling of NEpi in comparison to other cluster 1 tumors (Figure 6B). In mature cluster 2 PPGLs Epi was significantly elevated in comparison to immature cluster 1 tumors. Regression analysis demonstrated a significant correlation between the expression of *TH* and both *HIFα* subunits (Figure 6D,E).

## 3. Discussion

Hypoxia and the associated activation of hypoxia-related pathways contribute to tumor aggressiveness and therapy resistance [29,30]. As an alternative to extrinsic hypoxia, tumor cell spheroids provide a useful model to investigate the impact of hypoxia in vitro. Our study shows for the first time that the chromaffin cell features of pheochromocytoma cell lines remain unchanged during cultivation under spheroid conditions. This confirms the suitability of the model for investigations related to chromaffin cell features such as catecholamine biosynthesis, storage and secretion. For the tested cell lines, induction of spheroid formation via cultivation in the presence of methyl cellulose provided the most reproducible spheroids (Figures S1 and S2). Beside the described methods (Supplementary Materials), we also tested the agar-based liquid overlay technique [31,32,33] for all three pheochromocytoma cell lines. This method, however, resulted in multiple, small spheroids that were not useful for our purposes. The utilized methyl cellulose method is also suitable for the generation of endothelial cell spheroids [34]. This provides an opportunity for a future generation of multicellular spheroids consisting of pheochromocytoma cells, endothelial cells and/or fibroblasts providing a closer model to the in vivo situation within the tumor microenvironment.

**Figure 7:**
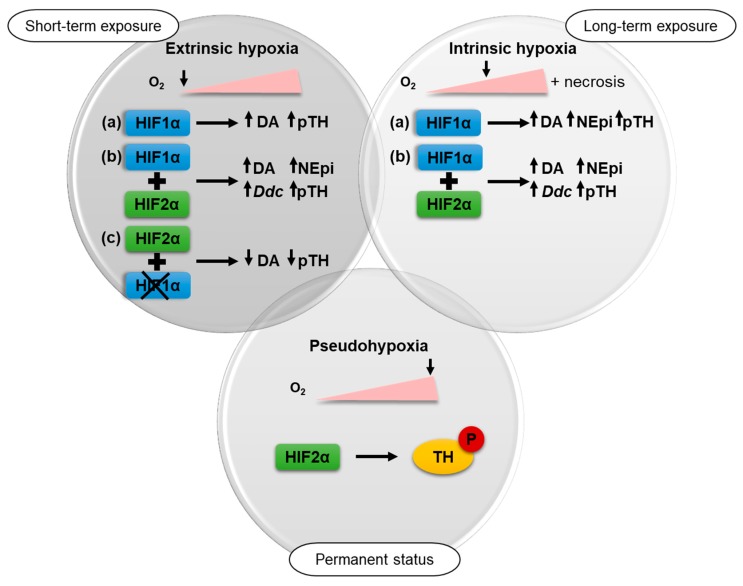
In the present study, the impact of three different types of hypoxia on the catecholamine biosynthesis of pheochromocytoma cells was investigated. Short-term exposure (24–48 h) to ≤ 1% oxygen under extrinsic hypoxia in *Hif2α*-deficient cells (**a**) led to an up-regulation of tyrosine phosphorylase (TH) phosphorylation and dopamine (DA). This effect was increased by long-term exposure to intrinsic hypoxia along with elevated norepinephrine (NEpi). Under both conditions similar effects could be observed after expression of *Hif2α* (**b**) in these cells. Expression of *Hif2α* further resulted in enhanced *Ddc* expression after hypoxic stimulation. The impact of extrinsic hypoxia could be reduced by a specific knockdown of *Hif1α* (**c**). Pseudohypoxia, characterized by permanent activation of hypoxia-related pathways in presence of oxygen, also led to an enhanced phosphorylation of TH.

Extrinsic as well as intrinsic hypoxia led to an up-regulation of total catecholamine contents of different pheochromocytoma cell lines with a cluster 2-like phenotype presumably reflecting increased phosphorylation of TH (Figure 7).

It is also possible that a reduced turnover of catecholamines due to influences on secretion or intracellular metabolism could also contribute to the increased catecholamine content under hypoxic conditions. Under spheroid conditions, an additional impact of necrosis on catecholamine turnover is conceivable. Different protein kinases and protein phosphatases mediate the phosphorylation of TH at serine residues Ser8, Ser19, Ser31 and Ser40. TH activity is nevertheless predominantly dependent on the phosphorylation at Ser40; phosphorylation at Ser31 also enhances TH activity but to a much lesser extent than for Ser40, and phosphorylation at Ser19 or Ser8 has no effect on the enzyme activity [13]. Protein kinase (PK) A, PKG, and PKC are primarily responsible for the phosphorylation of Ser40, whereas dephosphorylation is regulated by protein phosphatase 2A and 2C. Goldberg and coworkers showed that exposure to hypoxia led to an increased activation of different PKC isoforms [35]. A reactive oxygen species (ROS)-mediated activation of PKC was postulated as a mechanism for the increased secretory capacity of mouse adrenal chromaffin cells under chronic intermittent hypoxia [36]. Lee et al. documented a PKA-dependent effect on TH phosphorylation at Ser40, which seems to be responsible for elevated dopamine in PC12 cells under intermittent hypoxia [37]. In human cervical adenocarcinoma cells, hypoxia activated PKC-δ led to both increased HIF1α transcription and stability [38]. On the other hand, expression of PKA-α repressed the activity of HIF1α in PC12 cells [39]. The data of the present study further indicate an impact of Hif2α on TH phosphorylation. Expression of *Hif2α* in MTT and MPC-mCherry cells naturally lacking *Hif2α* led to an increased phosphorylation of TH under normoxic conditions. In further studies, it should be addressed if Hif2α-mediated phosphorylation of TH is directly or indirectly regulated by PKs independence on various hypoxic conditions (extrinsic, intrinsic and pseudohypoxic). The three tumors bearing a mutation in *EPAS1/HIF2α* consistently showed a doubling of NEpi in comparison to other cluster 1 PPGLs. For a final characterization of these tumors, the sample number needs to be increased. Differences in *TH* expression could not be observed. This suggests an increased phosphorylation of TH (Figure 6B) or reduced catecholamine turnover in vivo. Furthermore, expression of *Hif2α* in MPC-mCherry cells enhanced hypoxia-stimulated expression of *Ddc* (Figure 4). Brown and coworkers described two putative hypoxia response elements for HIF2α binding in the promoter region of *Ddc* [14]. This could be responsible for the enhanced *Ddc* expression in presence of *Hif1α* and *Hif2α*.

The present study allows for the first time a differentiation between HIF1α- and HIF2α-driven effects on the catecholamine biosynthesis under hypoxic conditions (Figure 7). The regulation of the catecholamine biosynthesis in pheochromocytoma cells seems to be primarily regulated by HIF1α under extrinsic hypoxia. A specific knockdown of *Hif1α* reduced hypoxia-induced dopamine synthesis and diminished TH phosphorylation significantly (Figure 5), while the expression of *Hif2α* had no additional effect (Figure 4). This is in line with findings of other groups showing that HIF2α is dispensable for the response to hypoxia [40,41]. Extrinsic hypoxia as performed in our study reflects only the effect of a short-term exposure (24–48 h) to reduced oxygen mainly leading in a stabilization of HIF1α. Tumor cell spheroids provide an excellent model to study long-term effects of hypoxia in vitro, but with considerations that results reflect a mixture of (a) proliferating cells under normoxia, (b) hypoxic cells, and (c) necrotic cells (Figure 1). In all cell lines cultivation under spheroid conditions increased cellular DA and NEpi contents compared to monolayer culture (Figure 2 and Figure 4), indicating an additional impact of necrosis. Immunohistochemical staining for TH showed an elevated expression in the necrotic core of the MTT and MPC spheroids (Figure 2). Little is known about the impact of necrosis on catecholamine biosynthesis, storage and turnover. Our data provide the first indication that necrosis enhances the biosynthesis of catecholamines in vitro.

The cellular catecholamine contents of pheochromocytoma cells in response to hypoxia seem to be primarily regulated through an increased phosphorylation of TH at Ser40. In short-term response (24–48 h), HIF1α regulates catecholamine biosynthesis, while HIF2α is dispensable for the direct response to hypoxia. A permanent activation of HIF2α, as shown in pseudohypoxic cluster 1 PPGLs for example, led to HIF2α-mediated phosphorylation of TH seen in MPC and MTT cells. Targeted inhibition of HIF2α possibly provides an excellent therapeutic approach for advanced PPGLs [42] and is moreover able to modulate catecholamine biosynthesis within the tumor cells.

## 4. Materials and Methods

If not indicated otherwise, all solutions and reagents were of the highest purity available from Sigma Aldrich GmbH (St. Louis, MO, USA). Cell culture medium and additives were obtained from Gibco (Thermo Fisher Scientific, Waltham, MA, USA) with the exception of fetal calf serum (Biowest, Riverside, MO, USA).

### 4.1. Cell Culture

Mouse pheochromocytoma cells (MPC 4/30/PRR) generated from heterozygous neurofibromatosis knockout mice and its more aggressive derivate termed MTT were used as models [24,25]. MPC cells were cultivated with RPMI-1640 containing 10% horse serum (HS), 5% fetal calf serum (FCS) and 2 mM Glutamax. For MTT cells Dulbecco’s Modified Eagle Medium (DMEM) + Glutamax supplemented with 10% HS, 5% FCS and 1 mM sodium pyruvate were used. For the cultivation of the PC12 rat pheochromocytoma cell line RPMI-1640 containing 10% horse serum (HS) and 5% fetal calf serum (FCS) were used [43]. All media were named complete medium in the following sections. All cell lines were acquired from Arthur Tischler (Department of Pathology and Laboratory Medicine, Tufts University School of Medicine, Boston, MA, USA and Karel Pacak. In general, cells were cultured at 37 °C, 5% CO_2_ and 95% humidity. MycoAlert Mycoplasma Detection Kit (Lonza, Basel, Switzerland) was used for testing cells to be mycoplasma free. After trypsinization (trypsin/EDTA; 0.05%/0.02%) cells were diluted with complete medium and counted by using C-CHIPs (Neubauer improved). Cultivation and all experiments were performed in absence of antibiotics. Cultivation and experiments in monolayer culture were performed using collagen A coated cell culture dishes. To generate extrinsic hypoxia, cells were cultivated under reduced oxygen partial pressure (≤1% oxygen) in an incubator furnished with an oxygen sensor (Sanyo InCuSafe O_2_/CO_2_ Incubator, Model MCO-5M, Osaka, Japan).

### 4.2. Hif2a Gene Knockin in MPC-mCherry and MTT Cells

MPC-mCherry H2A, expressing *Hif2α*, and their counterpart cell line MPC-mCherry control, transfected with an empty vector, were cultivated in collagen coated flask with antibiotic selection as previously described [22,44,45]. For the generation of *Hif2α* expressing MTT cells we used cells with stable expression of a non-targeting shRNA construct (SHC002V) [46]. These cells were transfected with pcDNA3.1+ carrying a codon-optimized version of the murine *Epas1* gene (MTT H2A; Genescript, Piscataway, NJ, USA) via nucleofection (4D-Nucleofector™ System, Lonza). At the same time, an empty-vector control cell line was generated (MTT control). Both cell lines were maintained after geneticin (250 µg/mL; Thermo Fisher Scientific) selection (Table S1). All experiments were performed in absence of antibiotics.

### 4.3. HIF1α Gene Knockdown in PC12 Cells

To achieve a specific HIF1α gene knockdown in PC12 cells rat HIF1α siRNA (sc-45919, Santa Cruz Biotechnology, Dallas, TX, USA) was used. A transfection efficiency of 57.9 ± 5.7% was achieved by using 12.5 nM siRNA per well in presence of lipofectamine RNAiMAX (Thermo Fisher Scientific). As control, aqua was utilized for transfection instead of siRNA.

### 4.4. Spheroid Generation

Cells were trypsinized from monolayer culture and an optimized cell number of 500 cells per spheroid were used for spheroid generation using the methyl cellulose (MC) method as previously described by us [28]. Additionally, two other methods for the generation of the spheroids were tested. A comparison of all three methods, (A) methyl cellulose method, (B) medium method, and (C) NunClon method, is shown in the Supplementary Materials. In general, spheroids were grown under standard culture conditions (5% CO_2_, 37 °C). Spheroid formation was considered as completed four days after seeding (Figure S1); thereafter medium was replaced by fresh complete medium with or without addition of 0.24% methyl cellulose after three to four days of cultivation. Spheroids were harvested 11 or 18 days after generation.

### 4.5. Protein Measurement

For measurements of protein, 500 cells were seeded in a 24-well plate (monolayer) or spheroids were generated as previously described. Spheroids and cells in monolayer culture were cultivated for eight days without changing medium for comparable conditions. After cultivation, two spheroids were combined in an Eppendorf tube. Spheroids and cells in monolayer culture were washed with PBS. Cell lysis was performed by using CellLytic^TM^ M with protease inhibitor. Protein amounts per spheroid or per well of 24-well plates were analyzed using the Bradford assay (Bio-Rad Laboratories, Hercules, CA, USA) according to manufacturer specifications.

### 4.6. Catecholamine Measurements

For measurements of catecholamines 12–24 spheroids were combined in an Eppendorf tube, washed in PBS and homogenized with at least five volumes of 0.4 M perchloric acid containing 0.5 mM ethylenediaminetetraacetic acid (50 µL) on ice. For cells in monolayer culture, 1 × 10^5^ cells were plated in a 24-well plate. After three days of cultivation, cells were washed and homogenized with 100 µL perchloric acid as described. All samples were centrifuged (1500× *g*, 15 min, 4 °C), supernatants were collected and catecholamines were analyzed by liquid chromatography with electrochemical detection as described previously [47]. Concentrations of catecholamines calculated relative to total protein. Therefore, a separate well of the 24-well plate or a separate Eppendorf tube containing the same number of spheroids was lysed and analyzed using the Bradford assay outlined above.

### 4.7. Hematoxylin and Eosin Staining

Spheroids were transferred in a Eppendorf tube, washed twice with ice-cold PBS and fixed with phosphate-buffered paraformaldehyde (4%) for 2 h. Fixed spheroids were stored in PBS containing 0.1% sodium azide (pH 7.2) at 4 °C. Spheroids were dehydrated with a series of processed alcohol ending with isopropanol and embedded in paraffin. For immunohistochemical or Hematoxylin and Eosin (H&E) staining 4-µm-thick sections were fixed on SuperFrost Plus slides and air-dried. Sections were deparaffinizedusing Neo-Clear and hydrated via descending ethanol series ending with 70% ethanol. For the H&E staining, sections were dyed with hematoxylin (Gill III), rinsed with 0.1% hydrochloric acid, differentiated with flowing water and stained with 0.5% aqueous eosin G solution. After rinsing in water, sections were rehydrated with processed alcohol series ending with 100% ethanol. Sections were mounted with Neo-Mount.

### 4.8. Immunohistochemistry

Paraffin sections were deparaffinized and hydrated as described above ending with distilled water. Spheroid sections were demarcated, washed with PBS and blocked for 1 h at room temperature with 1% bovine serum albumin in PBS containing 5% goat serum (blocking solution 1) followed by an overnight incubation at 4 °C with polyclonal rabbit anti-tyrosine hydroxylase (NB300-109, Novus Biologicals, Centennial, CO, USA, 1:100 in blocking solution 1). After incubation, sections were washed three times with PBS and incubated for 1 h with the secondary antibody Cy^TM^3 AffiniPure goat anti-rabbit IgG (111-165-144, Jackson Immunoresearch, Cambridgeshire, UK, 1:500 in PBS). After three washing steps with PBS, sections were counterstained with 4′,6-diamidino-2-phenylindole (DAPI; Sigma-Aldrich, 1:1000 in PBS) for 1.5 min, washed again with PBS and mounted with fluorescence mounting medium.

### 4.9. Pimonidazole Staining

Hypoxic areas within the spheroids were stained using the Hypoxyprobe-Kit (Hypoxyprobe, Burlington, MA, USA). Spheroids cultivated for 11 or 18 days were incubated with pimonidazole (20 µg/mL in phosphate-buffered saline, 30 min), fixed with formaldehyde, and embedded in paraffin. After dewaxing with RotiClear (Carl Roth GmbH & Co. KG, Karlsruhe, Germany) and rehydration in a graded series of ethanol solutions (100, 80, 70, 60, 50%, H_2_O) spheroids were de-masked via incubation in citric acid buffer (10 mmol/L, pH 6, 100 °C, 20 min). Thereafter endogenous peroxidase was blocked using hydrogen peroxide (3% in Tris-buffered saline, 10 min). Nonspecific binding sites were blocked for 1 h using blocking solution (10% fetal bovine serum (*v*/*v*) in Tris-buffered saline). Spheroid sections were incubated for 1 h with primary anti-pimonidazole antibody (PAb2627, Hypoxyprobe, 1:200 in blocking solution) and secondary biotinylated donkey anti-rabbit antibody (RPN1004V1, Amersham Biosciences, Little Chalfont, UK, 1:200 in blocking solution), respectively. For isotype control, spheroids were incubated with IgG rabbit (ab37415, Abcam, Cambridge, UK) instead of primary antibody. Specific binding was detected using extra-avidin-peroxidase (E2886, Sigma Aldrich, 1:50 in Tris-buffered saline, 30 min) followed by incubation with 3-Amino-9-ethylcarbazole (Thermo Fisher Scientific) and counterstaining with hematoxylin.

### 4.10. SDS-PAGE and Western Blot Analysis

HIF-1α, HIF-2α, PNMT, TH, pTH, and actin were analyzed by Western blot. After incubation under normoxic or hypoxic (≤1% O_2_) conditions, cells were washed with PBS, detached (typsin/EDTA) and resuspended in cold medium. After centrifugation at 4 °C, pellets were washed twice with PBS and stored at −80 °C. Sixty spheroids were transferred to each Eppendorf tube, washed three times with PBS and stored at −80 °C. Lysates were prepared on ice using CellLytic^TM^ M (Sigma-Aldrich, C2978) with protease inhibitors (1:100, Sigma-Aldrich; P8340). After 30 min incubation on ice and thoroughly mixing, cell lysates were centrifuged to remove cell debris. Protein concentration of all lysates were quantified using the Bradford assay. Fifty µg protein were mixed with LDS sample buffer (C.B.S. Scientific, San Diego, CA, USA; FB31010) and 5% mercaptoethanol. After denaturation at 99 °C for 5 min, proteins were separated on a 10% SDS-polyacrylamide gel and transferred to a polyvinylidine difluoride membrane (0.45 µm; Whatman, Buckinghamshire, UK) by semi-dry electroblotting. Non-specific binding sites on the membrane were blocked (5% skimmed milk powder plus 2% bovine serum albumin in TBS-T, blocking solution) at room temperature. Membranes were incubated with primary antibodies anti-PNMT (1:500; ab90862; abcam plc., Cambridge, UK), anti-tyrosine hydroxylase (1:1000, NB300-109, Novus Biologicals), anti-tyrosine hydroxylase phospho S40 (1:500, ab51206; abcam plc.), anti-HIF-1 alpha (1:200; NB10-479; Novus Biologicals), anti-HIF-2 alpha (1:200; NB10-479; Novus Biologicals), and anti-actin (1:1000; MAB1501R, Millipore, Massachusetts, USA) for 2 h at room temperature followed by an overnight incubation at 4 °C. After three washing steps in TBS-T, membranes were incubated for 1 h at room temperature with peroxidase-conjugated secondary antibody goat anti-rabbit IgG (1:5000; sc-2004; Santa Cruz Biotechnology) or goat anti-mouse IgG (1:5000; sc-2005; Santa Cruz Biotechnology). All antibodies were diluted in blocking solution. Protein visualization was performed as previously described [28].

### 4.11. Tumor Procurement and Genetic Testing

Snap frozen tumor tissue was collected directly after surgery from 25 patients with PPGL. Patients were enrolled in two different studies (Dresden/Germany, NIH/Bethesda/USA). All patients from Dresden are part of the PMT study (https://pmt-study.pressor.org/), ethic code EK 189062010; all patients from NIH were enrolled under the IRB Protocol 00-CH-0093. All patients have signed informed consent. Patients or tumor tissue were tested for germline and/or somatic mutations in established susceptibility by the CNIO institute in Madrid through a collaborative multi-center study (prospective monoamine-producing tumor study, https://pmt-study.pressor.org/) as previously described [48].

### 4.12. RNA Isolation and qRT-PCR

RNA from cells, spheroid pellets, or human PPGL tissue was isolated using RNeasy Plus Mini kit (Qiagen, Hilden, Germany) in accordance with manufacturer’s instructions. Reverse transcription of RNA and qRT-PCR was performed as described previously by us [28]. Sequence of each primer pair is summarized in detail in the supporting material (Table S2).

### 4.13. Statistical Analysis

Descriptive data are expressed as means ± SEM with statistical analyses taking into considerations numbers (*n*) of technical and biological replicates within independent experiments. Statistical analyses were carried out by one-way analysis of variance with post hoc Bonferroni tests using SigmaPlot 12.5 (Systat Software GmbH, Erkrath, Germany).

## 5. Conclusions

Our present study showed for the first time the suitability of pheochromocytoma spheroids to analyze the impact of hypoxia and necrosis on chromaffin cell features. Using either this model or extrinsic hypoxia in presence or absence of *Hif1α* or *Hif2α*, we demonstrated that Hif1α predominantly regulates catecholamines during short-term responses (24–48 h) to hypoxia, while Hif2α is dispensable for the direct response. Continuous activation of hypoxia-related genes under pseudohypoxic conditions leads to Hif2α-mediated activation of the catecholamine biosynthesis (permanent status).

## Supplementary Materials

The following are available online at https://www.mdpi.com/2072-6694/11/5/594/s1, Figure S1: MPC cell spheroid formation, Figure S2: MTT cell spheroid formation, Figure S3: Determining the optimal cell number to generate MTT cell spheroids, Figure S4: Growth curves and protein amount of MPC and MTT cell spheroids generated by three different methods, Figure S5: Pheochromocytoma cell spheroids stained with pimonidazole to visualize hypoxic regions, Figure S6: Impact of extrinsic and intrinsic hypoxia on catecholamine biosynthesis, Figure S7: Distribution of hexose-monophosphate (HMP) and phosphatidylinositol (PIP) in MTT cell spheroids, Table S1: Gene expression of MTT H2A cells compared to the MTT control cells was analyzed by reverse transcriptase polymerase chain reaction, Table S2: Primer sequences and targeted genes.
