# Supplementary material for: Impact of Extrinsic and Intrinsic Hypoxia on Catecholamine Biosynthesis in Absence or Presence of Hif2α in Pheochromocytoma Cells
Source: Cancers (Basel). 2019 Apr 28;11(5):594. doi: 10.3390/cancers11050594 (PMC6562431; doi:10.3390/cancers11050594)
Supplement: Supplementary file 1 [file cancers-11-00594-s001.pdf]

Supplementary Materials: Impact of Extrinsic and Intrinsic Hypoxia on Catecholamine Biosynthesis in Absence or Presence of Hif2α in Pheochromocytoma Cells

Nicole Bechmann, Isabel Poser, Verena Seifert, Christian Greunke, Martin Ullrich, Nan Qin, Axel Walch, Mirko Peitzsch, Mercedes Robledo, Karel Pacak, Jens Pietzsch, Susan Richter and Graeme Eisenhofer

1. Confirmation HIF2α Re-Expression MTT H2A Cells

Stable expression of codon-optimized version of the murine *Epas1* gene was confirmed via quantitative RT-PCR (Table S1). Primers recognize only the codon-optimized version not endogenous murine *Epas1*.

Table S1. Gene expression of MTT H2A cells compared to the MTT control cells was analyzed by reverse transcriptase polymerase chain reaction. Three independent experiments were performed (n = 3). Mean ± SEM. Cycle threshold (Ct).

Cell Line	Ct (*Hif2α*)	SEM	Ct (*actin*)	SEM
MTT control	0.0	0.0	17.1	0.4
MTT H2A	24.8	0.1	17.1	0.2

2. Experimental Details: Primer Quantitative RT-PCR

Table S2. Primer sequences and targeted genes.

Gene	Forward Primer Sequence	Reverse Primer Sequence	bp
	Human		
Human *TH*	GGAAGGCCGTGCTAAACCT	GGATTTTGGCTTCAAACGTCTC	99
Human *EPAS1*	GACCAATGCAGTACCCAGAC	GTGGCTGGAAGATGTTTGTC	184
Human *HIF1α*	GCTGATTTGTGAACCCATTC	AAATTGAGCGGCCTAAAAGT	164
Human *actin*	GGACTTCGAGCAAGAGATGG	AGCACTGTGTTGGCGTACAG	234
	Mouse		
Mouse *Dbh*	ACAGGTTCAGCAGTGAGGAG	CGGACAGCAGAAGTCTTGTT	157
Mouse *Ddc*	CCTGATTCCATTCTTTGTGG	ATCTGCAAACTCCACACCAT	187
Mouse *Th*	GGCTTCTCTGACCAGGCGTAT	TGCTTGTATTGGAAGGCAATCTC	67
Mouse *actin*	AAGGCCAACCGTGAAAAGAT	GTGGTACGACCAGAGGCATAC	110
	Rat		
Rat *Dbh*	TCTGTGCCCTGGAACTCTTT	CTGCGGAAGTGATCTTAGGC	145
Rat *Ddc*	CAGCTCCTACCCAGCTATGC	CAGCCAATCCATCATCACTG	118
Rat *Th*	TGTGTCCGAGAGCTTCAATG	GGGCTGTCCAGTACGTCAAT	120
Rat *Hif1α*	GTGGATTACCACAGCTGACC	CCCACAGACAACAACAACAA	178
Rat *Hif2α*	TCATCATGTGTGAGCCAATC	GTCATGTTCTCCGAGTCCAG	202
Rat *actin*	CACACTGTGCCCATCTATGA	CCGATAGTGATGACCTGACC	272

3. Spheroid Characterization and Method Comparison

For spheroid cultivation, three different methods were compared within the study to find optimal conditions and to investigate the impact of different cultivation methods on chromaffin cell features. In general, cells were trypsinized from monolayer culture and an optimized cell number of 500 cells per spheroid was used for spheroid generation with one of the three following methods. The methyl cellulose (MC) method (A) uses the addition of methyl cellulose stock solution to prevent

subsidence of cells as previously described by us [28]. For the medium method (B), cell suspensions without addition of methyl cellulose were seeded in cell culture plates for suspension culture (No.650185, Greiner Bio One, Kremsmünster, Austria). For the third method, termed the NunClon method (C), cell suspensions were added to Nunclon Sphera 96U-well plates (No. 174925, ThermoFisher Scientific, Waltham, MA, USA). Spheroids were grown under standard culture conditions (5% CO_2, at 37 °C). Spheroid formation was considered complete four days after seeding (Figure S1); thereafter medium was replaced by fresh complete medium with or without addition of 0.24% methyl cellulose after 3–4 days of cultivation. Spheroids were harvested 11 and 18 days after generation. No spheroid formation was achieved for PC12 cells using any of the three methods as well as the liquid overlay technique. Size of each spheroid was measured using the inverse microscope Axiovert 200M (Software: AxioVision 4.8, Zeiss, Göttingen, Germany). Area (A) of each spheroid was determined using the software package Fiji (ImageJ, Bethesda, MD, USA, www.imagej.net). Diameters (d) were calculated assuming an approximately spherical shape of the spheroids ($d = 2 \times \sqrt{(A/\pi)}$).

3.1. Spheroid Formation and Optimal Cell Number

Spheroid formation was monitored over a time period of four days via microscopy (Figures S1 and S2). Optimal cell numbers were evaluated for all three conditions by seeding 500–10,000 cells in each well of a 96-well plate with growth monitoring over 18 days (Figure S3). The optimal cell numbers for MPC and MTT cells were determined at 500 cells per spheroid. These numbers of cells were required to generate uniform single spheroids without detectable outgrowth over 18 days.

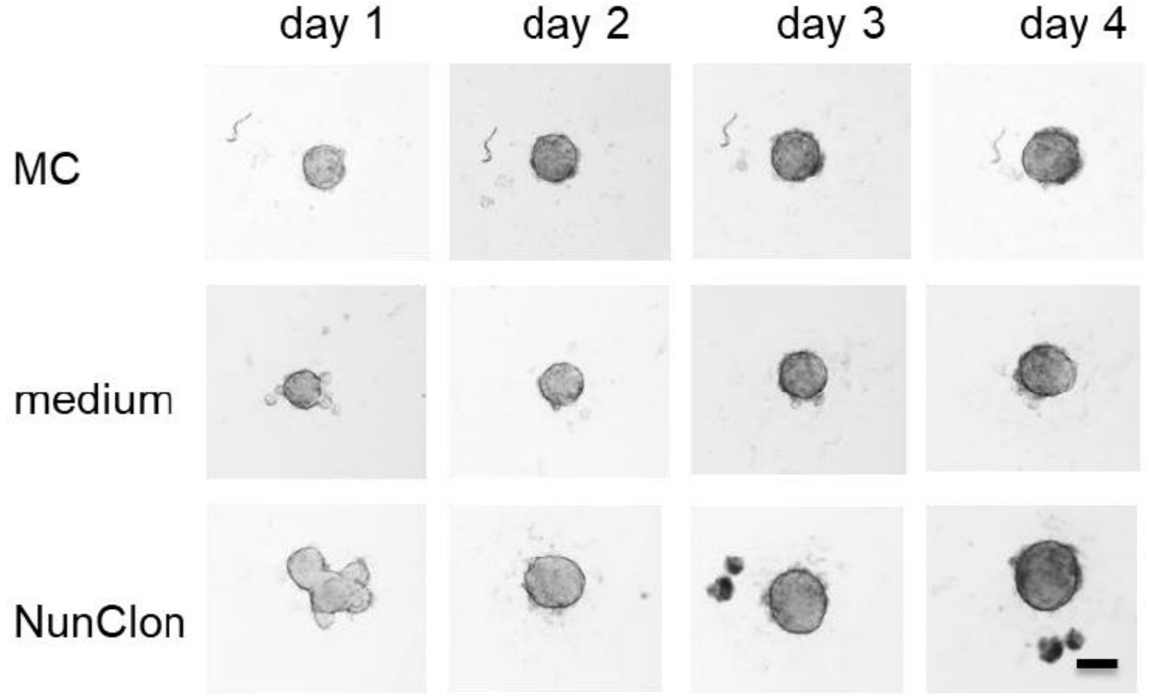

Figure S1. MPC cell spheroid formation. MPC cell spheroid formation has been monitored for four days. Only cultivation in presence of methyl cellulose provides spheroids with a smooth shape already after 24 h incubation. Scale bar: 100 μm.

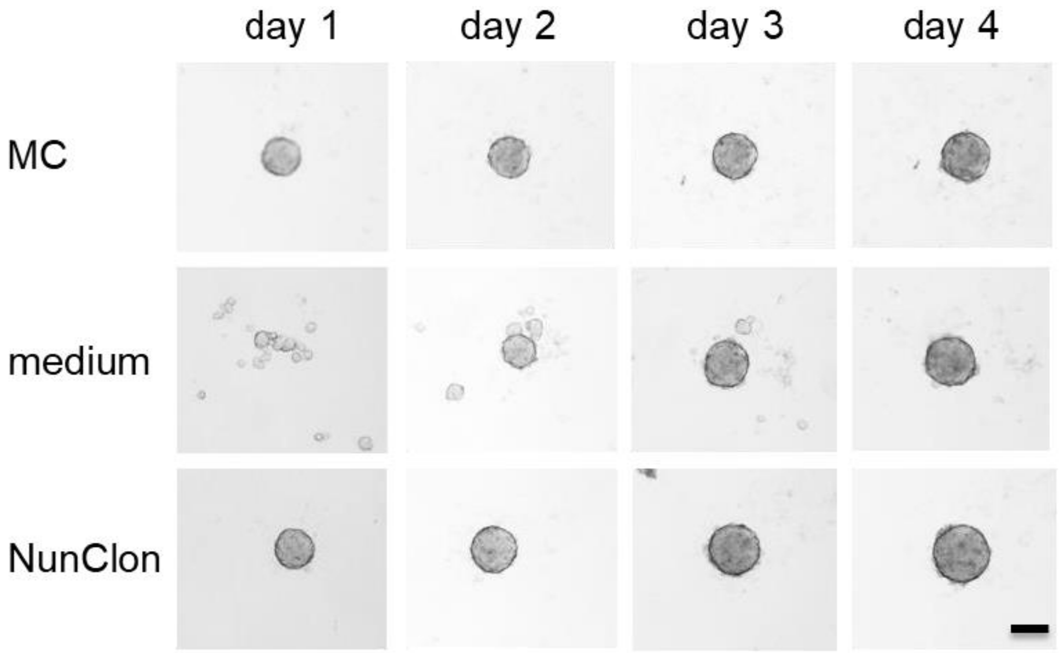

Figure S2. MTT cell spheroid formation. MTT cell spheroid formation has been monitored for four days. Cultivation in presence of methyl cellulose and using NunClon plate provides spheroids with a smooth shape already after 24 h incubation. Scale bar: 100 µm.

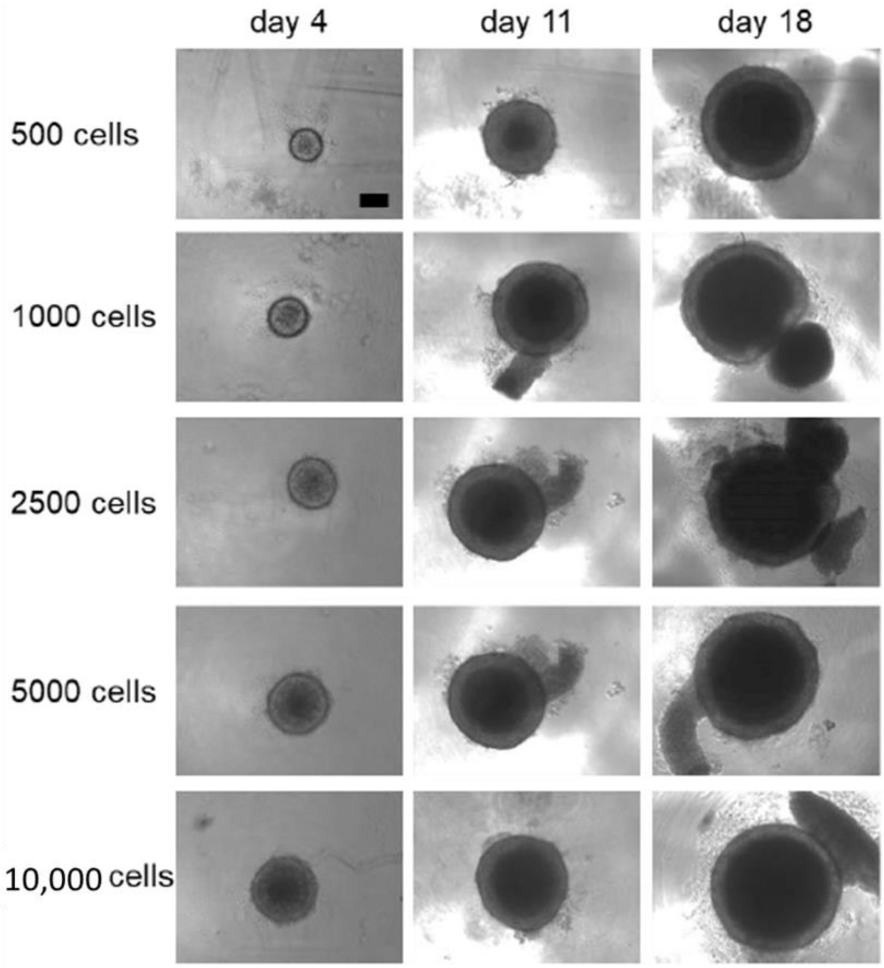

Figure S3. Determining the optimal cell number to generate MTT cell spheroids. In each well of an ultra-low binding 96-well plate (NunClon) 500–10,000 MTT cells were seeded and growth was monitored. For the other methods, similar results were obtained. Scale bar: 200 µm.

3.2. Spheroid Growth Pattern and Morphology

All three methods enabled formation of uniform, circular spheroids with uniform surface characteristics (Figure S4A,B) that were resistant to mechanical stress. The diameters of generated spheroids were comparable between all three methods. The total amount of protein in spheroids generated using NunClon plates was significantly lower compared to cultivation in monolayer culture (Figure S4C).

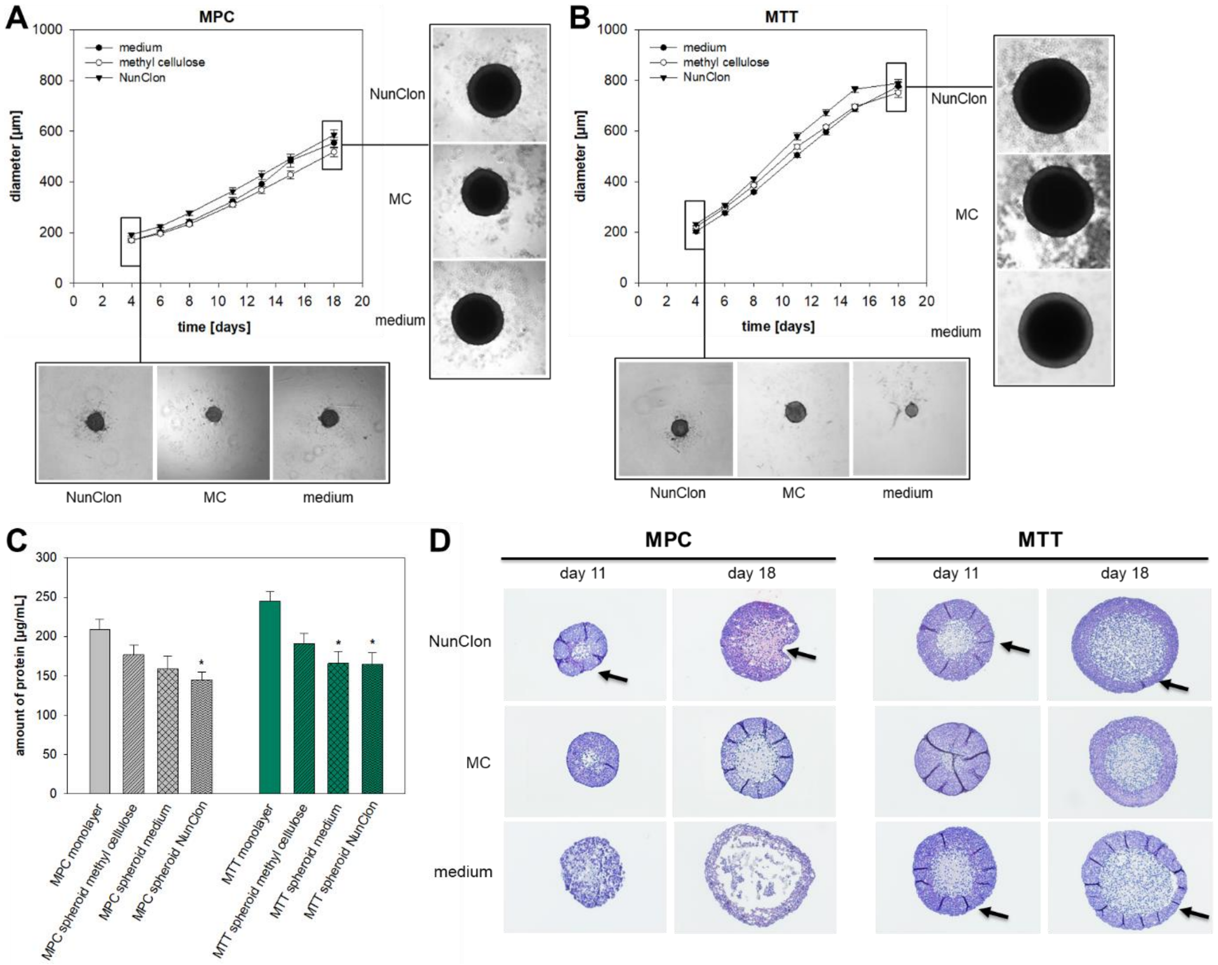

Figure S4. Growth curves and protein amount of MPC and MTT cell spheroids generated by three different methods. Impact of three different spheroid culturing conditions on the growth pattern of (**A**) MPC and (**B**) MTT cell spheroids. Four independent experiments (n = 15–20). Mean ± SEM. (**C**) Protein amount of 500 cells, as indirect tool to analyze the impact of different cell culture conditions, was analyzed after 8 days under monolayer or spheroid conditions. Cultivation under spheroid conditions resulted in decreased protein amount. Four independent experiments (n = 16). Mean ± SEM. ANOVA and Bonferroni *post hoc* test comparison vs. monolayer, * $p < 0.05$ or, ** $p < 0.01$. (**D**) Representative section of MPC and MTT cell spheroids stained with Hematoxylin and Eosin. Arrows mark irregularities in the proliferating cell layer thickness. Scale bar: 200 µm.

These results indicate that the NunClon plates generate less compact spheroids, a result of their larger size but associated with reduced total cell material. The trend was that generation of spheroids was associated with reduced amounts of protein, which can be explained by the structure of spheroids. As a consequence of the diminished oxygen and nutrient supply a necrotic core surrounded by a hypoxic ring was formed within spheroids (Figures 1 and S5). Only outer cell layers are able to proliferate. Subsequently proliferation is reduced in comparison to monolayer culture. The necrotic area developed within 11 days (Figure S4D) in both cell lines. Hematoxylin and Eosin staining showed considerable differences between different methods that could be related to various

section planes. Spheroids cultivated with the medium methods showed a more diffuse structure especially in MPC cell spheroids. Spheroids generated using the NunClon plates demonstrated a solid structure but the outer proliferating cell layer seems to be inconsistent with regard numbers of cell layers (marked with an arrow, Figure S4D). In comparison, spheroids generated using methyl cellulose showed a solid structure and uniform distribution of the proliferating outer cell layer in both cell lines.

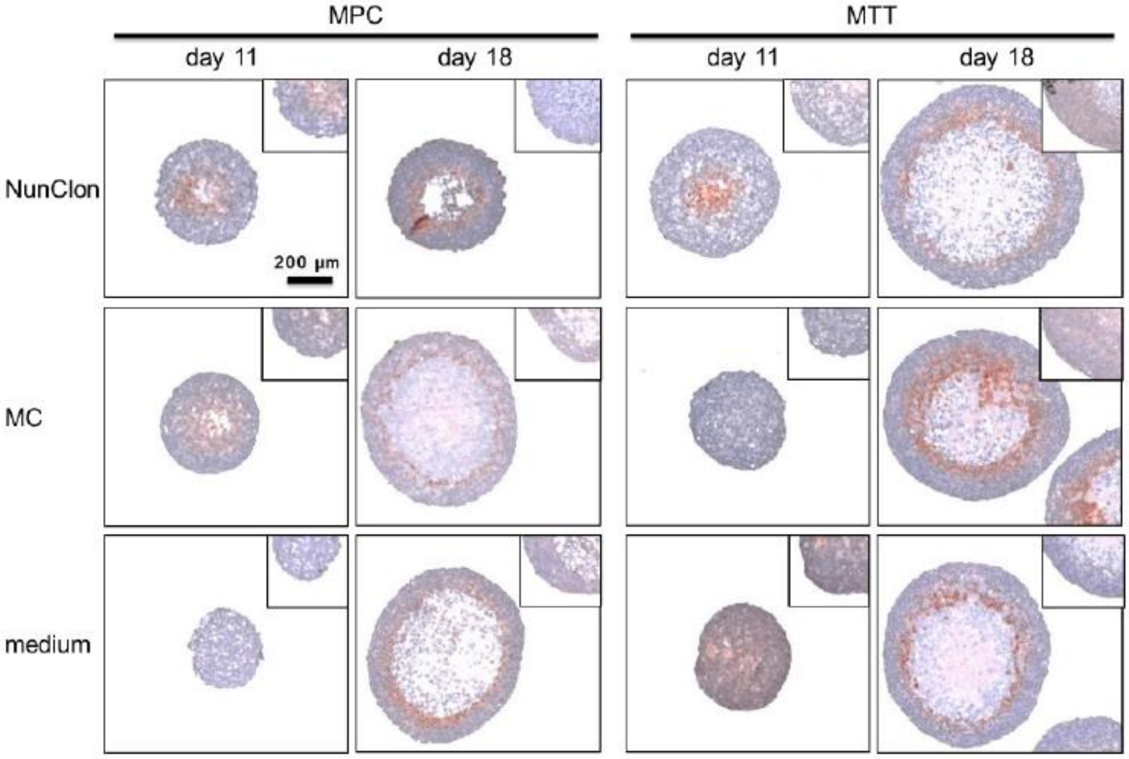

Figure S5. Pheochromocytoma cell spheroids stained with pimonidazole to visualize hypoxic regions. To visualize hypoxic regions within the spheroid pimonidazole was used as hypoxia marker. In comparison to 18 days old spheroids, 11 days old spheroids showed a pimonidazole localization in the spheroid core. Once spheroids get bigger (18 days of cultivation), the hypoxic area surrounded the necrotic core clearly. Representative picture of three independent experiments.

A characteristic of chromaffin cells involves the biosynthesis of catecholamines. Both cell lines produced dopamine (DA) and norepinephrine under monolayer conditions. Cellular levels of DA were noticeably increased under extrinsic hypoxia. All three conditions for spheroid cultivation resulted in a significant increase in cellular DA contents (Figure S6A,B). Cultivation under MC and medium conditions lead to significantly increased DA contents after 18 days cultivation in comparison to day 11. This effect was not detectable using the NunClon method. Cultivation under extrinsic and intrinsic conditions resulted in a strengthened phosphorylation of tyrosine hydroxylase (TH), whereas TH protein levels remained unaffected (Figure S6C).

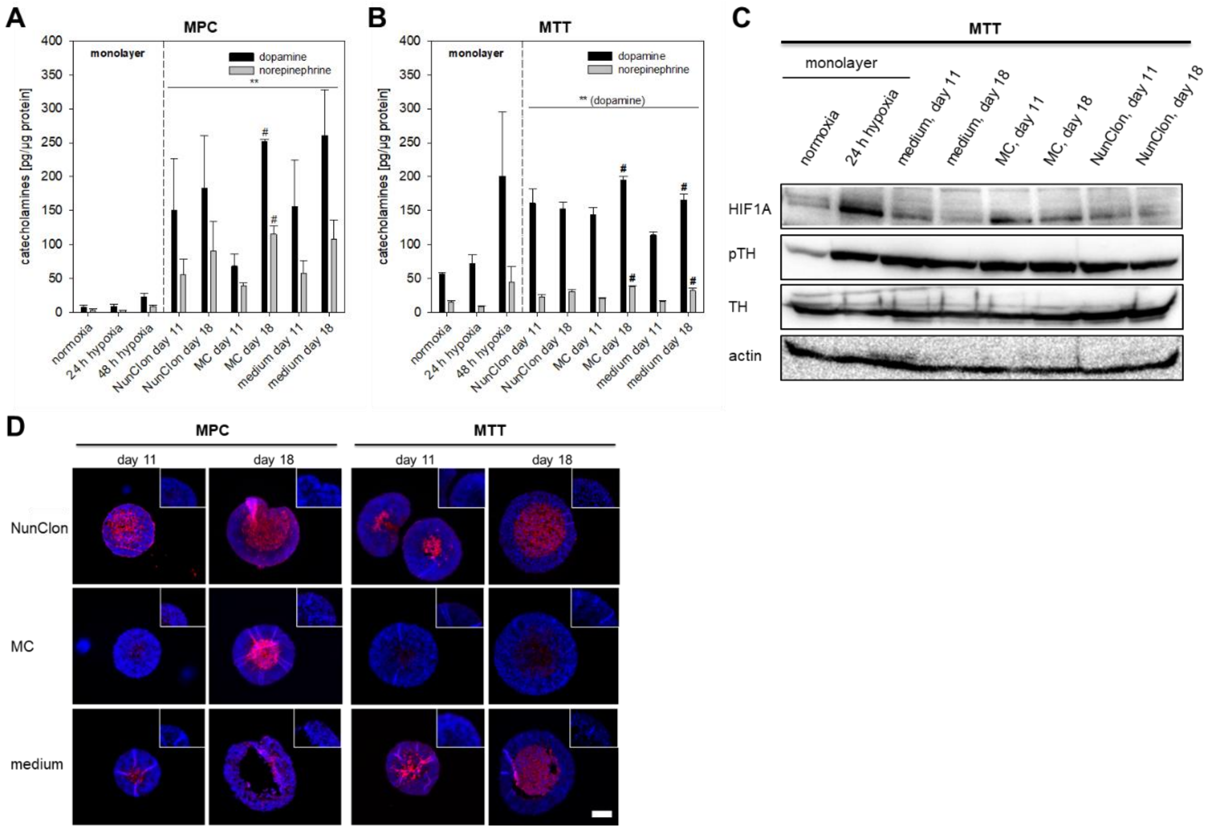

Figure S6. Impact of extrinsic and intrinsic hypoxia on catecholamine biosynthesis. Catecholamine storage of (**A**) MPC and (**B**) MTT cell spheroids in comparison to monolayer cultivation under normoxic or hypoxic conditions. Three independent experiments (n = 3–6). Mean ± SEM. ANOVA and Bonferroni *post hoc* test comparison vs. normoxia, * $p < 0.05$ or, ** $p < 0.01$, or vs. spheroid day 11, # $p < 0.05$ or, ## $p < 0.01$. (**C**) Effect of extrinsic and intrinsic hypoxia on protein synthesis of catecholamine converting enzymes. Representative section of three independent western blot analyses. (**D**) Immunohistochemical staining of tyrosine hydroxylase (red) in paraffin sections of the spheroid counterstained with DAPI (blue), scale bar: 200 µm.

4. MALDI Mass Spectrometry Imaging (MALDI-MSI) of Pheochromocytoma Spheroids

4.1. Methods

4.1.1. Cryosectioning of Spheroids

Spheroids were embedded and fresh-frozen in a water drop to prepare cryosections with a thickness of 14 µm at −20 °C using a Crytostat (CM1950, Leica), and were mounted on indium tin oxide (ITO)-coated glass slides (Bruker Daltonik GmbH, Bremen, Germany) pretreated with 1:1 poly-L-lysine (Sigma-Aldrich, Munich, Germany) and 0.1% Nonidet P-40 (Sigma-Aldrich).

4.1.2. Sample Preparation for MALDI-MSI Analysis

1,5-Diaminonaphthalene (DAN) (Cat.-No. 56451-250MG, Sigma-Aldrich) was used as MALDI matrix. Briefly, a 10 mg/mL stock solution of DAN in 70% acetonitrile was prepared and filtered through a syringe driven filter (0.2 µm, PTFE). The matrix was applied using the SunCollect system (SunChrom) using the following protocol: In total, eight layers were sprayed with different flow rates: Layer 1: 10 µL/min; Layer 2: 20 µL/min; Layer 3: 30 µL/min and Layers 4 to 8: 40 µL/min.

4.1.3. MALDI-MSI Measurements

MALDI-MSI measurements were performed on a solariX 7 T FTICR mass spectrometer (Bruker, Billerica, MA, USA) in the mass range of *m*/*z* 50–1000 in negative ion mode with an estimated resolution of 35,000 at *m*/*z* 400 and a raster width of 50 µm at a frequency of 1000 Hz, using 100 laser shots, and "small" laser focus. Spectra were recorded on Compass solariXcontrol software with 1 M data points, a transient length of 0.26 s, and an ion cooling time of 0.01 s. Source conditions were as follows: deflector plate at −220 V, funnel 1 at −150 V, skimmer 1 at −15 V, and funnel rf amplitude of 150 Vpp. Calibration of the mass spectrometer was conducted using L-arginine in the electrospray ionization (ESI) mode. Two non-tissue measurement regions were included as background controls, one in the beginning and another in the end of the measurement. After MALDI-MSI, matrix was removed with 70% ethanol, stained with Hematoxylin and Eosin, mounted with a coverslip, scanned with a Mirax Desk scanner (Zeiss, Göttingen, Germany) with 20-fold magnification objective, and co-registered to respective MSI data using FlexImaging (version 4.0) (Bruker, Billerica, MA, USA). FlexImaging (Bruker, Billerica, MA, USA, www.bruker.com) was also used to analyze the ion maps and for the export of images.

4.2. Distribution of Selected Metabolites within MTT Cell Spheroids

Two different sections of an MTT cell spheroid were analyzed with regard to distribution of hexose-monophosphate (HMP) and phosphatidylinositol (PIP). Hematoxylin and Eosin staining were used to confirm the cut in the proliferating cell zone (first section) or in the middle of the spheroid (second section), displaying the necrotic and hypoxic core surrounded by proliferating cells (Figure S7). Originally, this analysis was designed to measure the distribution of catecholamines within spheroids, but dopamine, norepinephrine and epinephrine contents within the spheroid were below the MALDI/MS imaging technique's limit of detection.

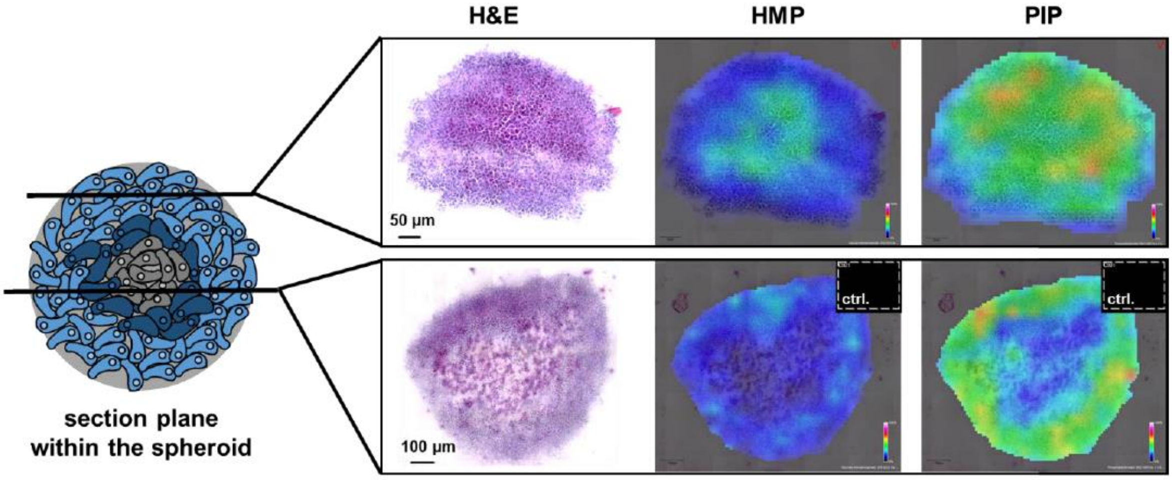

Figure S7. Distribution of hexose-monophosphate (HMP) and phosphatidylinositol (PIP) in MTT cell spheroids. Two different sections of the MTT cell spheroid were generated via cryosectioning. Hematoxylin and Eosin staining confirmed the cross section in the proliferating cell layer (upper pictures) while the second section (lower pictures) showed a cross section of the necrotic/hypoxic core surrounded by a proliferating cell zone. MALDI-MSI records of HMP and PIP, demonstrated an accumulation of these metabolites only in the proliferating cell zone.

The activity of the hexose monophosphate shunt, also known as pentose phosphate pathway, was analyzed. This pathway occurs in parallel to glycolysis and generates NADPH, pentoses and ribose 5-phosphate. A higher activity in outer cell layers confirmed that more activity is shunted in this pathway. In comparison, the inner core of the spheroids showed no HMP activity. Phosphatidylinositol and its phosphorylated derivatives form a minor component of the cell

membrane. Higher levels of PIP in the proliferating cell layers indicate that the membrane of the cells remained intact, while PIP intensity is reduced as expected under necrotic conditions.

© 2019 by the authors. Licensee MDPI, Basel, Switzerland. This article is an open access article distributed under the terms and conditions of the Creative Commons Attribution (CC BY) license (http://creativecommons.org/licenses/by/4.0/).
